# Role of endogenous ouabain in the etiology of bipolar disorder

**DOI:** 10.1186/s40345-020-00213-1

**Published:** 2021-02-01

**Authors:** Rif S. El-Mallakh, Yonglin Gao, Pan You

**Affiliations:** 1 Mood Disorders Research Program, Depression Center, Department of Psychiatry and Behavioral Sciences, University of Louisville School of Medicine, 401 East Chestnut Street, Suite 610, Louisville, KY 40202 USA; 2 Xiamen Xianyue Hospital, 399 Xianyue Road, Xiamen, China

**Keywords:** Bipolar disorder, Calcium, Endogenous ouabain, Ouabain, Pathophysiology, Sodium, Sodium pump

## Abstract

**Background:**

Bipolar disorder is a severe psychiatric illness with poor prognosis and problematic and suboptimal treatments. Understanding the pathoetiologic mechanisms may improve treatment and outcomes.

**Discussion:**

Dysregulation of cationic homeostasis is the most reproducible aspect of bipolar pathophysiology. Correction of ionic balance is the universal mechanism of action of all mood stabilizing medications. Recent discoveries of the role of endogenous sodium pump modulators (which include ‘endogenous ouabain’) in regulation of sodium and potassium distribution, inflammation, and activation of key cellular second messenger systems that are important in cell survival, and the demonstration that these stress-responsive chemicals may be dysregulated in bipolar patients, suggest that these compounds may be candidates for the coupling of environmental stressors and illness onset. Specifically, individuals with bipolar disorder appear to be unable to upregulate endogenous ouabain under conditions that require it, and therefore may experience a relative deficiency of this important regulatory hormone. In the absence of elevated endogenous ouabain, neurons are unable to maintain their normal resting potential, become relatively depolarized, and are then susceptible to inappropriate activation. Furthermore, sodium pump activity appears to be necessary to prevent inflammatory signals within the central nervous system. Nearly all available data currently support this model, but additional studies are required to solidify the role of this system.

**Conclusion:**

Endogenous ouabain dysregulation appears to be a reasonable candidate for understanding the pathophysiology of bipolar disorder.

## Background

Bipolar disorder is a severe psychiatric illness that manifests as extreme variations in mood and energy, usually labelled as mania and depression, interspersed over an euthymic or dysthymic baseline (Ketter and Calabrese 2002). The disorder afflicts approximately 1% of people (Clemente et al. (2015); Grande et al. 2016), with documented suboptimal treatments and a host of undesirable outcomes related to both the disease and its treatment (Cipriani et al. 2017; Dome et al. 2019). Despite over 60 years of directed effort, the pathoetiology of the illness remains unknown (Grande et al. 2016). Nonetheless, multiple clues have emerged that continue to inform ongoing research. The illness is viewed as multifactorial with elements of development and neuroplasticity, inflammation, and aberrant modulation of brain function and circuitry, that are mediated by gene and environment interaction through multiple inherited genes and multiple altered epigenetic changes (Grande et al. 2016; Belvederi Murri et al. 2016; Nestler et al. 2016; Takaesu 2018). Due to the absence of a centralized unifying model, pathophysiologic research continues in a fragmented, siloed fashion. A proposed mechanism of pathophysiology that incorporates much of the translational and clinical data, might help focus and thus accelerate research efforts.

Among the most reproducible findings in bipolar illness has been dysregulation of control of electrically important ions: sodium (Na^+^), potassium (K^+^), hydrogen (proton, H^+^), and calcium (Ca^2+^) (El-Mallakh et al. 1076). Ion regulation spans across all of the proposed mechanisms of pathogenesis of abnormal moods in bipolar illness and across all successful treatment options. For example, in an analysis of susceptibility loci, Askland found that approximately 74% of known loci involve genes related to ion regulation, or what she refers to as neuroelectrical genes (Askland 2006). By comparison, genes involved in any neurotransmitter pathway account for only about 58% of the susceptibility loci, and the monoamines account to only 31% (10). A similar conclusion is reached when genome wide association studies (GWAS) are explored (Judy and Zandi 2013). The only animal models of bipolar illness that meet all validity criteria create ion transport abnormalities (Mack et al. 2019; Valvassori et al. 2019). Additionally, nearly all interventions that are effective in mania or mood stabilization reduce intracellular sodium either directly or indirectly (El-Mallakh and Huff 2001; El-Mallakh and Paskitti 2001; Roberts et al. 2010). A model in which a primary ion abnormality can produce the symptoms of bipolar illness has been proposed (El-Mallakh and Wyatt 1995) but the question remains how are environmental stressors that may lead to mood recurrence translated into pathophysiologic abnormalities that cause mood symptoms?

**Fig. 1:**
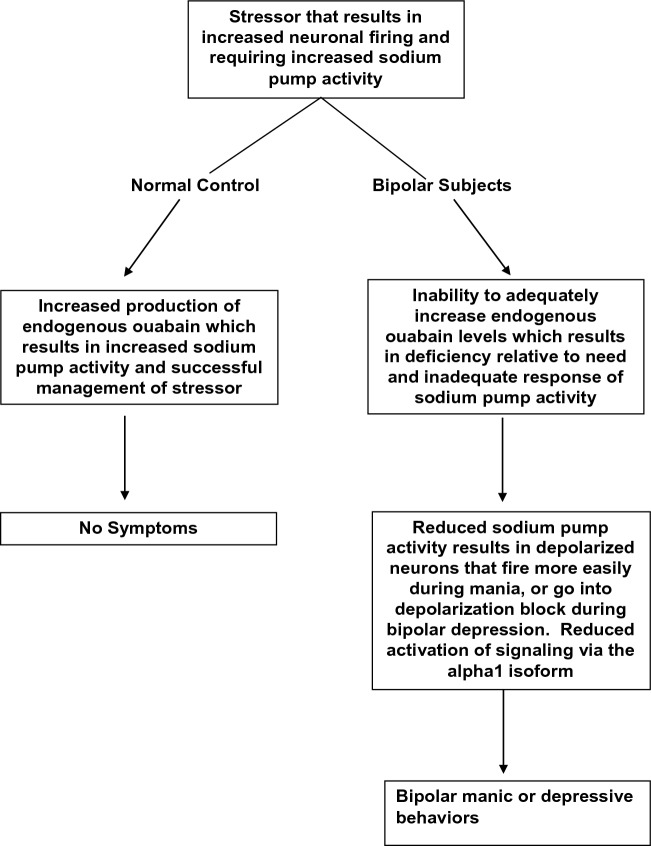
Proposed model of role of endogenous ouabain in pathophysiology of bipolar disorder

The authors propose that endogenous cardiac steroids may be the factors that transduce the consequences of stressors to produce syndromes of mania or depression via abnormal neural function (Fig. 1). This paper will review the evidence that endogenous cardiac steroids, such as endogenous ouabain, may be involved in the pathogenesis of bipolar illness, and how these stress-responsive compounds may lead to periods of illness.

## Methods

PubMed and Google Scholar were interrogated with the search words of “digoxin mood,” “digoxin bipolar,” “ouabain mood,” “ouabain bipolar,” “cardenolide mood,” and “cardenolide bipolar.” Only the first 100 titles of each search were reviewed by two authors, because the utility declined quickly after that, and articles that informed the topic were specifically read.

## Results

The possible involvement of ion dysregulation and endogenous cardiac steroids in BD was recently reviewed (El-Mallakh et al. 1076; Mack et al. 2019; Valvassori et al. 2019; Lichtstein et al. 2018). “Digoxin mood” yielded 507 items on Pubmed and 11,000 on Google Scholar. “Digoxin bipolar” yielded 17 items on Pubmed and 9720 on Google Scholar. Similarly, “ouabain mood” yielded 1288 and 580, respectively; and “ouabain bipolar” yielded 108 and 5890. “Cardenolide mood” and “cardenolide bipolar” yielded 1329, 230, 101, and 216 items.

## Discussion

### Endogenous cardiac steroids and the sodium pump

Endogenous cardiac steroids include digoxin-like, ouabain-like, and bufadienolide-like molecules. These molecules were first identified in plants (Foxglove, *Digitalis,* for digoxin (Kaul et al. 2013), twisted flower, *Strophanthus*, for ouabain (Fürstenwerth 2019)) and toads (*Rhinella* and other for bufadienolides (Krenn and Kopp 1998; Abdelfatah et al. 2019)). These agents have been used for centuries for treating congestive heart failure, atrial fibrillation, and appear to have a positive inotropic effect on cardiac muscle (Norn and Kruse 2004).

Cardiac steroids effects are mediated by multiple mechanisms. They are known to bind to specific sites on the α subunit of the sodium and potassium-activated adenosine triphosphatase (Na,K-ATPase) or sodium pump. The discovery of the sodium pump resulted in a 1997 Noble Prize to Jens C. Skou, the Danish scientist who documented its existence in 1957 (Skou (1957)). It is usually composed of two subunits, α and β. The α subunit has the sodium, potassium, adenosine triphosphatase (ATP), magnesium, and cardiac glycoside binding sites, while the β subunit is needed for membrane localization of the catalytic αsubunit and cellular adhesion. There are four different isoforms of the α subunit, and mRNA of all four can be found in nearly all human tissues. Protein expression of α1 isoform is ubiquitous in all human tissues. The α2, α3, and α4 are found in human brain, heart, and to a lesser extent, breast and reproductive tissues (GeneCards Human Gene Database 2020). The pump is additionally regulated by a small peptide that binds to the β subunit that was initially called γ, but is now referred to as FXYD domain containing ion transport regulator (Geering 2005). FXYD1 in muscle and FXYD2 in the kidney generally reduce sodium pump activity, FXYD7 in the brain also does so by reducing the affinity of the potassium binding site to extracellular potassium (Geering 2005; Clausen et al. 2020).

In the last 20 years, acceptance that cardiac glycosides are synthesized in the adrenal and hypothalamus of mammals has increased (El-Masri et al. 2002; Lichtstein et al. 1998; Blaustein 2018), but is still not universal (Baecher et al. 2014). It is known that cholesterol (which may be reduced in the brains of patients with bipolar illness (Beasley et al. 2005)) is needed for endogenous ouabain production (Blaustein 2018); and the pathway may involve pregnenolone and progesterone as intermediate steps, (Baecher et al. 2014). (Interestingly, pregnenolone levels may be reduced in the cerebrospinal fluid of individuals with a diagnosis of mood disorder *and* in relation to the severity of their symptoms (George et al. 1994).) Once synthesized, endogenous bufadienolides in toads (Lichtstein et al. 1993; Butler et al. 1996), and endogenous cardenolides in mammals (Antolovic et al. 1998) may circulate bound to a plasma protein. Rapid elevations in plasma levels of endogenous cardiac glycosides, as occurs with exhaustive exercise (Valdes et al. 1988), may occur due to release of bound glycosides from this carrier protein (Antolovic et al. 2000). The structure of the endogenous glycosides strongly resembles their plant-derived or toad-derived namesakes (Goto et al. 1989; Hamlyn et al. 1991; Komiyama et al. 2000). There is a large and reproducible literature that finds the endogenous glycosides play a significant role in regulation of fluid and electrolytes in animals and humans (Hamlyn and Manunta 2011) and have been associated with a multitude of diseases that involve expanded volume (El-Mallakh et al. 2019).

### Physiological actions of endogenous glycosides

Plants manufacture glycosides for the purpose of protection. The concentrations of glycosides in plant tissues are toxic to animals and reduce herbivory. That means that at the concentrations in plants glycosides are at superphysiologic concentrations. Different glycosides have different actions. For example, marinbufagenin, which has higher affinity with preferential inhibition of the α1 isoform (Feraille and Doucet 2001; Dvela et al. 2007) and greater constriction of vascular smooth muscle (Dvela et al. 2007). Administration of ouabain may increase blood pressure, while digoxin would actually lower it (Huang et al. 1999; Manunta et al. 2000), and endogenous compounds appear to have a similar effect (Balzan et al. 2007). For understanding bipolar disorder, endogenous ouabain appears to be most important (El-Mallakh et al. 2010).

At concentrations found in plants or used experimentally, glycosides inhibit the activity of the Na, K-ATPase and have served as the major experimental purpose of cardenolides, particularly ouabain (Noël et al. 2018). Recently it has been demonstrated that in physiologic mammalian fluids the concentrations of endogenously produced ouabain are quite low (El-Mallakh et al. 2010; Manunta et al. 2006; Dvela et al. 2012). At these physiologic picomolar to nanomolar concentrations glycosides *increase* sodium pump activity in heart, smooth muscle, kidney, and brain (Ghysel-Burton and Godfraind 1979; Gao et al. 2002; Dvela-Levitt et al. 2015; Lichtstein et al. 1985). Even when disease increases the plasma levels of endogenous ouabain, the total levels remain below concentrations that inhibit sodium pump activity (Holthouser et al. 2010). The ouabain-induced increase of sodium pump activity is related to the affinity of the glycoside-binding site to the endogenous ligand, so that α3 appears more susceptible to inhibition with lower concentrations, followed by α2 and α1 at progressively higher concentrations (Balzan et al. 2007; Gao et al. 2002; Holthouser et al. 2010; Saunders and Scheiner-Bobis 2004). This effect may not be a direct effect on the sodium pump but may be secondary to signal transduction on α1, and possibly α 3, and α 4. Specifically, ouabain may increase sodium pump activity through activation of Src kinase-, ERK1/2-, and Akt-mediated pathway or the sodium-proton exchanger 1 (NHE1) (Holthouser et al. 2010; Liang et al. 2006; Khundmiri et al. 2007, 2008).

More recently, it has been discovered that endogenous glycosides can activate second messenger signals through phosphorylation of the Src protein (Thomas and Brugge 1997; Cui and Xie 2017). This discovery has been very exciting because it ties in endogenous glycosides with many cell regulatory processes that occur at physiologic, low nM concentrations of endogenous ouabain (Lichtstein et al. 2018; Touza et al. 2011). The effect on cellular signaling may be of particular importance for the pathophysiology of mood disorders, in general, and bipolar disorder, in particular (Lichtstein et al. 2018). Specifically, activation of extracellular signal-regulated kinase (ERK), protein kinase B (AKT), and nuclear factor kappa-light-chain-enhancer of activated B cells (NFkB) result from the interaction of endogenous ouabain with its receptor on the α 1 isoform which phosphorylate the Src intracellular protein (Lichtstein et al. 2018). These systems are important in calcium signaling and neurotransmitter regulation (Lichtstein et al. 2018). ERK and Raf–mitogen-activated protein kinase (MAPK) are involved in cell survival and proliferation (Kim and Choi 2010), which may be important in cell loss in bipolar patients (Chen and Manji 2006; Yuan et al. 2010; Schroeder et al. 2016).

Activation of the sodium pump appears to be an essential feature to reduce central nervous system inflammation (Kinoshita et al. 2017). Sterile inflammation appears to be a characteristic of bipolar disorder pathophysiology (Rosenblat and McIntyre 2016). If sodium pump activity is blocked in glial cells, inflammatory pathways are activated in the presence of lipopolysaccharides (Kinoshita et al. 2017).

Because the sodium pump activating effect of ouabain occurs at physiologic (low) concentrations (Holthouser et al. 2010), this effect is probably more important to understanding physiologic actions of endogenous ouabain than studies using pharmacologic (high) doses of the plant-derived form of this compound.

The concentrations of endogenous ouabain in the brain may be particularly important in understanding the differential roles of control of ion regulation and control of signal transduction. The α1 isoform is clearly involved in signal transduction (Cui and Xie 2017; Madan et al. 2017), even though all isoforms have highly conserved sequences important in regulating that interaction (Cui and Xie 2017). Alpha2 does not appear to activate ERK, but α3 and α4 appear to do so with at lower doses of ouabain (Pierre et al. 2008). In studies in which cells are designed to express only the α3 isoform, the Src protein does not appear to be phosphorylated, but the ERK system is still activated (Madan et al. 2017), which may occur due to increases of intracellular calcium or other disruption of cellular function (Balasubramaniam et al. 2015; Ghilardi et al. 2020). Similarly, mice without a functional α3 protein show increased calcium signaling in cultured cortical neurons and phospho-activation of ERK and Akt in the hippocampus (Kirshenbaum et al. 2011a), suggesting that increased intracellular calcium may play a role in signal transduction independent of α3. If these differences in isoform involvement in the Src/ERK/MAPK signaling pathway apply in human brains, and given that the affinity of α3 glycoside receptor site greatly exceeds that of α1 (Pierre et al. 2008), activation of ion transport activity will occur via α3 before activation via α1.

### Glycoside induced behavior changes in animal models

Creating an animal model for psychiatric illnesses is always difficult. Bipolar illness may be particularly hard since an adequate animal model would manifest both mania *and* depression *and* response *and* prevention of both with medications like lithium (Fineberg et al. 2011). The only animal models of mania that achieve face validity, construct validity, and predictive validity are ionic models (Mack et al. 2019). One of these models utilizes administration of high doses of ouabain by intracerebroventricular (ICV) injection in rats (El-Mallakh et al. 2003).

Direct ICV ouabain administration will reduce sodium pump activity in the brain (Hamid et al. 2009), induce both depressive and manic symptoms which can be treated and prevented with lithium (Valvassori et al. 2019; El-Mallakh et al. 1995; Li et al. 1997). The model attempts to mimic the reduced sodium pump activity that has been reported in bipolar illness, not changes in endogenous ouabain. The outcome of both a deficiency of endogenous ouabain (which is expected to increase Na, K-ATPase activity), or an excess of exogenous ouabain (which reduces Na, K-ATPase activity), would be expected to be the same—a reduction in sodium pump activity.

However, there are animal models of depression that suggest that reducing sodium pump activity may be antidepressive. Specifically, ICV administration of anti-ouabain antibodies to normal rats has been shown to reduce depressive behaviors (Goldstein et al. 2006). Additionally, in animal models of depression such as Flinders Sensitive Line of genetically sensitized rats to diisopropyl fluorophosphate (DFP, which inhibit organophosphorus cholinesterase (Overstreet 1993)), or lipopolysaccharide-treated rats, treatment with ICV anti-ouabain antibodies also improves depressive symptoms (Goldstein et al. 2006, 2012). Similarly, a three-fold increase of brain endogenous glycoside occurs in the amphetamine model of mania, but the hyperactivity is prevented with ICV administration of anti-ouabain antibodies (Hodes et al. 2016).

It is not clear which brain area and how many degrees of the reduction of sodium pump activity are associated with manic or depressive behaviors.

### Endogenous ouabain aberration in bipolar disorder subjects

Before the discovery of the sodium pump in 1957, John Cade in Australia described the antimanic effect of lithium treatment (Cade 1949). By the mid-1950s the efficacy of lithium treatment had been well-established in Europe (Schou et al. 1954; Rice 1956). This created an initial focus on ion regulation in bipolar illness and led to early studies in this area. Most importantly were a large number of studies that documented a mood-state related reduction in sodium pump activity (Looney and El-Mallakh 1997). These changes were seen in both bipolar depression and mania. Furthermore, whole body intracellular sodium was also increased in both mania and depression and normalizes with treatment or euthymia (Coppen et al. 1966; Shaw 1966; Coppen 1967). Since intracellular sodium concentration is a major determinant of intracellular calcium concentrations (Blaustein 1993), it is not surprising that free intracellular calcium is also elevated in mania and normalizes with treatment or euthymia (Dubovsky et al. 1989, 1992). Postmortem brain studies of the sodium pump in patients with bipolar illness report that the expression of the α2 isoform is reduced in the temporal cortex of patients with bipolar illness compared to non-bipolar controls (Rose et al. 1998), and the expression of the α3 subunit is increased overall but reduced in GABAergic neurons in several brain regions of patients with bipolar illness versus non-psychiatrically ill controls (Hodes et al. 2019). There are specific haplotypes of the α2 subunit that have been associated with bipolar illness in a small sample of unrelated subjects (Goldstein et al. 2009). In the periphery, regulation of the α1 of both red cells (Looney and El-Mallakh 1997; Nurnberger et al. 1982) and immortalized white cells (Huff et al. 2010; Li and El-Mallakh 2004; Cherry and Swann 1994) is impaired in bipolar individuals. These findings served as some of the basis of the Na, K-ATPase hypothesis for bipolar illness, in which it is proposed that increases of these ions depolarized neurons and make them more likely to fire and lead to mania, while more substantial increases of intracellular sodium would lead to depolarization block and depression (El-Mallakh and Wyatt 1995). Genetic associations to both the α1 and α3 subunit genes of the Na, K-ATPase have been reported (Goldstein et al. 2009; Mynett-Johnson et al. 1998), but have not been reliably reported (Philibert et al. 2001). The later study examined a population of Old Order Amish individuals that have been genetically isolated for nearly two centuries (Philibert et al. 2001; Crowley 1978). Animal models, which can be used to test hypotheses, reveal that heterozygote α2 knockout (KO) mice may have a partial manic-like picture [108, but heterozygote α3 KO mice have a more complete bipolar-like picture with both manic (Kirshenbaum et al. 2011a,^ 2014^, 2011b). These data are compatible with the interpretation that the decline of sodium pump activity associated with abnormal moods in bipolar patients is secondary to another process, such as differential elaboration of endogenous ouabain.

A non-specific antibody for endogenous glycosides was used to demonstrate that these agents are present in reduced concentrations in manic individuals versus normal controls (Li and El-Mallakh 2004). Furthermore, subjects with bipolar illness lacked the seasonal variation in circulating glycosides found in controls (low in winter and higher during the rest of the year), with similar levels during winter (the most stable time for bipolar patients) but lower levels for the rest of the year (Li and El-Mallakh 2004). These findings suggested that bipolar illness may be characterized by inability to upregulate endogenous ouabain production in response to environmental needs. Since endogenous glycoside synthesis is known to increase in response to exercise to exhaustion (Valdes et al. 1988), an experiment was performed with bipolar patients and non-athletic controls. Euthymic patients with bipolar illness did not increase endogenous ouabain levels when exercised to exhaustion compared to non-bipolar controls (El-Mallakh et al. 2010). This may have led to a reduced exercise duration of patients with bipolar illness (Cherry and Swann 1994). All of these measures were in peripheral blood; in postmortem brain tissue, endogenous glycoside levels were higher in the parietal cortex of patients who had bipolar illness compared to those with major depressive disorder and non-mentally ill controls, but not compared to those with schizophrenia (Goldstein et al. 2006). However, tissue measurements are limited by the fact that they are expressed per unit amount of protein, and so endogenous glycosides may appear higher in a group of patients whose illness manifests in tissue loss (Mynett-Johnson et al. 1998). Moreover, different brain tissues may have different affinity of endogenous ouabain binding, also it is possible that endogenous ouabain concentration of brain and peripheral blood may not synchronously change.

Release of endogenous ouabain from the adrenals appears to be under the control of adrenocortical trophic hormone (ACTH) (Philibert et al. 2001; Crowley 1978). Hypothalamic-pituitary axis (HPA) disruption is associated with ill phases of bipolar illness (Belvederi Murri et al. 2016) as are stressors that may predispose to affective recurrence, such as sleep deprivation (Gao et al. 2013). Sleep deprivation in mice is associated with increased corticosterone and endogenous ouabain (Gao et al. 2013). Similarly, experimental sleep deprivation increases cortisol levels and manic symptoms in men (Kirshenbaum et al. 2014). Even 24 h of sleep deprivation is enough to increase serum cortisol (Kirshenbaum et al. 2011b; Grider et al. 1999; Shah et al. 2007), though this finding is not universal (Moorhead et al. 2007). Rats that receive anti-ouabain antibodies microinjections into the locus coeruleus have a significant reduction in rapid eye movement (REM) sleep (Goto et al. 1996). Conversely, rats undergoing REM deprivation had a decrease in affinity to ouabain (Hinson et al. 1998). ICV ouabain enhanced wakefulness in rats (Gao et al. 2017) which is consistent with its manic-like effects (El-Mallakh et al. 2003, 1995; Hamid et al. 2009; Li et al. 1997), but did not induce any other lasting changes. No studies measuring endogenous glycosides in humans after sleep deprivation have been performed. However, it is expected that circulating endogenous ouabain would increase parallel to cortisol in humans. If observations that individuals with bipolar illness are unable to upregulate endogenous ouabain in response to need are correct, then one would predict, that sleep deprivation in bipolar subjects would not result in endogenous ouabain secretion.

### Proposed endogenous ouabain model of pathogenesis of bipolar disorder

Endogenous ouabain has the highest affinity of all the endogenous glycosides to the α3 isoform of the sodium pump. The α3 isoform has its greatest distribution in neurons in the central nervous system. At physiologic concentrations, endogenous ouabain increases sodium pump activity, and may or may not activate cellular signaling through the α1 isoform receptor. Under certain circumstances that include season of year and exhaustive exercise (and possibly sleep deprivation), endogenous ouabain levels increase in non-bipolar subjects, but do not appear to respond in people with bipolar illness (Fig. 1). The hypothesis argues that these are stressors in which increased sodium pump activity is needed, and since physiologic concentrations of endogenous ouabain increase pump activity, increased ouabain production is needed to deal with the stress. Patients with bipolar illness are not able to upregulate endogenous ouabain production, and so enter the stress with a relative deficiency of endogenous ouabain, resulting in a relative reduction in sodium pump activity, and excessively depolarized resting potential, and expected abnormalities in neural function that result in both mania and bipolar depression.

## Conclusions

Recent work has highlighted the potential role of endogenous glycosides, particularly endogenous ouabain-like factor, in the pathophysiology and possible pathogenesis of bipolar illness. It is purported that individuals with bipolar disorder appear to be unable to upregulate endogenous ouabain under conditions mandating the action of that hormone. This leaves patients with neural tissues that is unable to maintain adequate depolarization, and leads to symptoms of mania or bipolar depression. Nearly all available data currently support this model, but additional studies are required to solidify the role of this system. Endogenous ouabain dysregulation appears to be a reasonable candidate for understanding the pathophysiology of bipolar disorder.

## Data Availability

Not applicable.

## Abbreviations

ACTH: Adrenocortical trophic hormone
AKT: Protein kinase B
ATP: Adenosine triphosphatase
DFP: Diisopropyl fluorophosphate
ERK: Extracellular signal-regulated kinase
GABA: Gamma-Aminobutyric acid,
HPA: Hypothalamic-pituitary axis
ICV: Intracerebroventricular
Na,K-ATPase: Sodium- and potassium-activated adenosine triphosphatase
NFkB: Nuclear factor kappa-light-chain-enhancer of activated B cells
NHE1: Sodium-proton exchanger 1
MAPK: Mitogen-activated protein kinase

## References

[CR1] Abdelfatah S, Lu X, Schmeda-Hirschmann G, Efferth T (2019). Cytotoxicity and antimitotic activity of *Rhinella schneideri* and *Rhinella marina* venoms. J Ethnopharmacol.

[CR2] Antolovic R, Kost H, Mohadjerani M, Linder D, Linder M, Schoner W (1998). A specific binding protein for cardiac glycosides exists in bovine serum. J Biol Chem.

[CR3] Antolovic R, Bauer N, Mohadjerani M, Kost H, Neu H, Kirch U, Grünbaum EG, Schoner W (2000). Endogenous ouabain and its binding globulin: effects of physical exercise and study on the globulin's tissue distribution. Hypertens Res.

[CR4] Askland KD (2006). Toward a biaxial model of “bipolar” affective disorders: further exploration of genetic, molecular and cellular substrates. J Affect Disord.

[CR5] Baecher S, Kroiss M, Fassnacht M, Vogeser M (2014). No endogenous ouabain is detectable in human plasma by ultra-sensitive UPLC-MS/MS. Clin Chim Acta.

[CR6] Balasubramaniam SL, Gopalakrishnapillai A, Gangadharan V, Duncan RL, Barwe SP (2015). Sodium-calcium exchanger 1 regulates epithelial cell migration via calcium-dependent extracellular signal-regulated kinase signaling. J Biol Chem.

[CR7] Balzan S, D'Urso G, Nicolini G, Forini F, Pellegrino M, Montali U (2007). Erythrocyte sodium pump stimulation by ouabain and an endogenous ouabain-like factor. Cell Biochem Funct.

[CR8] Beasley CL, Honer WG, Bergmann K, Falkai P, Lütjohann D, Bayer TA (2005). Reductions in cholesterol and synaptic markers in association cortex in mood disorders. Bipolar Disord.

[CR9] Belvederi Murri M, Prestia D, Mondelli V, Pariante C, Patti S, Olivieri B, Arzani C, Masotti M, Respino M, Antonioli M, Vassallo L, Serafini G, Perna G, Pompili M, Amore M (2016). The HPA axis in bipolar disorder: systematic review and meta-analysis. Psychoneuroendocrinology.

[CR11] Blaustein MP (1993). Physiological effects of endogenous ouabain: control of intracellular Ca^2+^ stores and cell responsiveness. Am J Physiol.

[CR12] Blaustein MP (2018). The pump, the exchanger, and the holy spirit: Origins and 40-year evolution of ideas about the ouabain-Na^+^ pump endocrine system. Am J Physiol Cell Physiol.

[CR13] Butler VP, Morris JF, Akizawa T, Matsukawa M, Keating P, Hardart A, Furman I (1996). Heterogeneity and lability of endogenous digitalis-like substances in the plasma of the toad, *Bufo marinus*. Am J Physiol.

[CR14] Cade JF (1949). Lithium salts in the treatment of psychotic excitement. Med J Aust.

[CR15] Chen G, Manji HK (2006). The extracellular signal-regulated kinase pathway: an emerging promising target for mood stabilizers. Curr Opin Psychiatry.

[CR16] Cherry L, Swann AC (1994). Cation transport mediated by Na+, K(+)-adenosine triphosphatase in lymphoblastoma cells from patients with bipolar I disorder, their relatives, and unrelated control subjects. Psychiatry Res.

[CR17] Cipriani G, Danti S, Carlesi C, Cammisuli DM, Di Fiorino M (2017). Bipolar disorder and cognitive dysfunction: a complex link. J Nerv Ment Dis.

[CR18] Clausen MV, Hilbers F, Poulsen H (2017). The structure and function of the Na, K-ATPase isoforms in health and disease. Front Physiol.

[CR19] Clemente AS, Diniz BS, Nicolato R, Kapczinski FP, Soares JC, Firmo JO, Castro-Costa É (2015). Bipolar disorder prevalence: a systematic review and meta-analysis of the literature. Braz J Psychiatry.

[CR20] Coppen A (1967). The biochemistry of affective disorders. Br J Psychiatry.

[CR21] Coppen A, Shaw DM, Malleson A, Costain R (1966). Mineral metabolism in mania. Br Med J.

[CR22] Crowley WK (1978). Old Order Amish settlement: diffusion and growth. Ann Assoc Am Geographers.

[CR23] Cui X, Xie Z (2017). Protein interaction and Na/K-ATPase-mediated signal transduction. Molecules.

[CR25] Dome P, Rihmer Z, Gonda X (2019). Suicide risk in bipolar disorder: a brief review. Medicina.

[CR26] Dubovsky SL, Christiano J, Daniell LC, Franks RD, Murphy J, Adler L, Baker N, Harris RA (1989). Increased platelet intracellular calcium concentration in patients with bipolar affective disorders. Arch Gen Psychiatry.

[CR27] Dubovsky SL, Murphy J, Thomas M, Rademacher J (1992). Abnormal intracellular calcium ion concentration in platelets and lymphocytes of bipolar patients. Am J Psychiatry.

[CR28] Dvela M, Rosen H, Feldmann T, Nesher M, Lichtstein D (2007). Diverse biological responses to different cardiotonic steroids. Pathophysiology.

[CR29] Dvela M, Rosen H, Ben-Ami HC, Lichtstein D (2012). Endogenous ouabain regulates cell viability. Am J Physiol Cell Physiol.

[CR30] Dvela-Levitt M, Ben-Ami HC, Rosen H, Ornoy A, Hochner-Celnikier D, Granat M, Lichtstein D (2015). Reduction in maternal circulating ouabain impairs offspring growth and kidney development. J Am Soc Nephrol.

[CR31] El-Mallakh RS, Huff MO (2001). Mood stabilizers and ion regulation. Harv Rev Psychiatry.

[CR32] El-Mallakh RS, Paskitti ME (2001). The ketogenic diet may have mood-stabilizing properties. Med Hypoth.

[CR33] El-Mallakh RS, Wyatt RJ (1995). The Na, K-ATPase hypothesis for bipolar illness. Biol Psychiatry.

[CR34] El-Mallakh RS, Yff T, Gao Y. Ion Dysregulation in the pathogenesis of bipolar illness. Ann Depress Anxiety 2016;3(1):1076. https://austinpublishinggroup.com/depression-anxiety/fulltext/depression-v3-id1076.php.

[CR35] El-Mallakh RS, Harrison LT, Li R, Changaris DG, Levy RS (1995). An animal model for mania: preliminary results. Prog Neuro-Psychopharmacol Biol Psychiatry.

[CR36] El-Mallakh RS, El-Masri MA, Huff MO, Li X-P, Decker S, Levy RS (2003). Intracerebroventricular administration of ouabain to rats models human mania. Bipolar Disord.

[CR37] El-Mallakh RS, Stoddard M, Jortani SA, El-Masri MA, Sephton S, Valdes R (2010). Aberrant regulation of endogenous ouabain-like factor in bipolar subjects. Psychiatry Res.

[CR38] El-Mallakh RS, Brar KS, Yeruva RR (2019). Cardiac glycosides in human physiology and disease: update for entomologists. Insects.

[CR39] El-Masri MA, Clark BJ, Qazzaz HM, Valdes R (2002). Human adrenal cells in culture produce both ouabain-like and dihydroouabain-like factors. Clin Chem.

[CR40] Feraille E, Doucet A (2001). Sodium-potassium-adenosine triphosphatase-dependent sodium transport in the kidney: Hormonal control. Physiol Rev.

[CR41] Fineberg NA, Chamberlain SR, Hollander E, Boulougouris V, Robbins TW (2011). Translational approaches to obsessive-compulsive disorder: from animal models to clinical treatment. Br J Pharmacol.

[CR42] Fürstenwerth H (2019). Comment on endogenous ouabain and related genes in the translation from hypertension to renal diseases, International Journal of Molecular Science 2018;19:1948. Int J Mol Sci.

[CR43] Gao J, Wymore RS, Wang Y, Gaudette GR, Krukenkamp IB, Cohen IS, Mathias RT (2002). Isoform-specific stimulation of cardiac Na/K pumps by nanomolar concentrations of glycosides. J Gen Physiol.

[CR44] Gao Y, Jhaveri M, Lei Z, Chaneb BL, Lingrel J, El-Mallakh RS (2013). Glial-specific gene alterations associated with manic behaviors. Int J Bipolar Disord.

[CR45] Gao Y, Akers B, Roberts MB, El-Mallakh RS (2017). Corticosterone response in sleep deprivation and sleep fragmentation. J Sleep Disord Manage.

[CR24] GeneCards Human Gene Database. https://www.genecards.org/Search/Keyword?queryString=atp1a1. Accessed 10 Oct 2020.

[CR46] Geering K (2005). Function of FXYD proteins, regulators of Na. K-ATPase J Bioenerg Biomembr.

[CR47] George MS, Guidotti A, Rubinow D, Pan B, Mikalauskas K, Post RM (1994). CSF neuroactive steroids in affective disorders: pregnenolone, progesterone, and DBI. Biol Psychiatry.

[CR48] Ghilardi SJ, O'Reilly BM, Sgro AE (2020). Intracellular signaling dynamics and their role in coordinating tissue repair. Wiley Interdiscip Rev Syst Biol Med.

[CR49] Ghysel-Burton J, Godfraind T (1979). Stimulation and inhibition of the sodium pump by cardioactive steroids in relation to their binding sites and their inotropic effects on guinea-pig isolated atria. Br J Pharmacol.

[CR50] Goldstein I, Levy T, Galili D, Ovadia H, Yirmiya R, Rosen H, Lichtstein D (2006). Involvement of Na^+^, K^+^-ATPase and endogenous digitalis-like compounds in depressive disorders. Biol Psychiatry.

[CR51] Goldstein I, Lerer E, Laiba E, Mallet J, Mujaheed M, Laurent C, Rosen H, Ebstein RP, Lichtstein D (2009). Association between sodium- and potassium-activated adenosine triphosphatase alpha isoforms and bipolar disorders. Biol Psychiatry.

[CR52] Goldstein I, Lax E, Gispan-Herman I, Ovadia H, Rosen H, Yadid G, Lichtstein D (2012). Neutralization of endogenous digitalis-like compounds alters catecholamines metabolism in the brain and elicits anti-depressive behavior. Eur Neuropsychopharmacol.

[CR53] Goto A, Yamada K, Ishii M, Yoshioka M, Ishiguro T, Eguchi C, Sugimoto T (1989). Existence of a polar digitalis-like factor in mammalian hypothalamus. Biochem Biophys Res Commun.

[CR54] Goto A, Yamada K, Hazama H, Uehara Y, Atarashi K, Hirata Y, Kimura K, Omata M (1996). Ouabain like compound in hypertension associated with ectopic corticotropin syndrome. Hypertension.

[CR55] Grande I, Berk M, Birmaher B, Vieta E (2016). Bipolar disorder. Lancet.

[CR56] Grider G, El-Mallakh RS, Huff MO, Buss TJR, Miller J, Valdes R (1999). Endogenous digoxin-like immunoreactive factor (DLIF) serum concentrations are decreased in manic bipolar patients compared to normal controls. J Affect Disord.

[CR57] Hamid H, Gao Y, Lei Z, Hougland MT, El-Mallakh RS (2009). Effect of ouabain on sodium pump alpha-isoform expression in an animal model of mania. Prog Neuro-Psychopharmacol Biol Psychiatry.

[CR58] Hamlyn JM, Manunta P (2011). Endogenous ouabain: a link between sodium intake and hypertension. Curr Hyperten Rep.

[CR59] Hamlyn JM, Blaustein MP, Bova S, DuCharme DW, Harris DW, Mandel F, Mathews WR, Ludens JH. Identification and characterization of a ouabain-like compound from human plasma. Proc Natl Acad Sci USA. 1991;88(14):6259–6263. Erratum in: Proc Natl Acad Sci USA 1991;88(21):9907. 10.1073/pnas.88.14.6259 PMC52062 1648735

[CR60] Hinson JP, Harwood S, Dawnay AB (1998). Release of ouabain-like compound (OLC) from the intact perfused rat adrenal gland. Endocr Res.

[CR61] Hodes A, Rosen H, Deutsch J, Lifschytz T, Einat H, Lichtstein D (2016). Endogenous cardiac steroids in animal models of mania. Bipolar Disord.

[CR62] Hodes A, Rosen H, Cohen-Ben Ami H, Lichtstein D (2019). Na^+^, ^K^+-ATPase a3 isoform in frontal cortex GABAergic neurons in psychiatric diseases. J Psychiatr Res.

[CR63] Holthouser KA, Mandal A, Merchant ML, Schelling JR, Delamere NA, Valdes R, Tyagi SC, Lederer ED, Khundmiri SJ (2010). Ouabain stimulates Na-K-ATPase through a sodium/hydrogen exchanger-1 (NHE-1)-dependent mechanism in human kidney proximal tubule cells. Am J Physiol Renal Physiol.

[CR65] Huang BS, Kudlac M, Kumarathasan R, Leenen FH (1999). Digoxin prevents ouabain and high salt intake-induced hypertension in rats with sinoaortic denervation. Hypertension.

[CR66] Huff MO, Li XP, Ginns E, El-Mallakh RS (2010). Effect of ethacrynic acid on the sodium- and potassium-activated adenosine triphosphatase activity and expression in Old Order Amish bipolar individuals. J Affect Disord.

[CR68] Judy JT, Zandi PP (2013). A review of potassium channels in bipolar disorder. Front Genet.

[CR69] Kaul S, Ahmed M, Zargar K, Sharma P, Dhar MK (2013). Prospecting endophytic fungal assemblage of *Digitalis lanata* Ehrh. (foxglove) as a novel source of digoxin: a cardiac glycoside. 3 Biotech.

[CR70] Ketter TA, Calabrese JR (2002). Stabilization of mood from below versus above baseline in bipolar disorder: a new nomenclature. J Clin Psychiatry.

[CR71] Khundmiri SJ, Amin V, Henson J, Lewis J, Ameen M, Rane MJ, Delamere NA (2007). Ouabain stimulates protein kinase B (Akt) phosphorylation in opossum kidney proximal tubule cells through an ERK-dependent pathway. Am J Physiol Cell Physiol.

[CR72] Khundmiri SJ, Ameen M, Delamere NA, Lederer ED (2008). PTH-mediated regulation of Na^+^-K^+^-ATPase requires Src kinase-dependent ERK phosphorylation. Am J Physiol Renal Physiol.

[CR73] Kim EK, Choi E-J (2010). Pathological roles of MAPK signaling pathways in human diseases. Biochim Biophys Acta.

[CR74] Kinoshita PF, Yshii LM, Orellana AMM, Paixão AG, Vasconcelos AR, de Sá Lima L, Kawamoto EM, Scavone C (2017). Alpha 2 Na+, K+-ATPase silencing induces loss of inflammatory response and ouabain protection in glial cells. Sci Rep..

[CR75] Kirshenbaum GS, Clapcote SJ, Duffy S, Burgess CR, Petersen J, Jarowek KJ, Yücel YH, Cortez MA, Snead OC, Vilsen B, Peever JH, Ralph MR, Roder JC (2011). Mania-like behavior induced by genetic dysfunction of the neuron-specific Na^+^, ^K^+-ATPase a3 sodium pump. Proc Natl Acad Sci USA.

[CR76] Kirshenbaum GS, Saltzman K, Rose B, Petersen J, Vilsen B, Roder JC (2011). Decreased neuronal Na^+^, ^K^+-ATPase activity in* Atp1a*3 heterozygous mice increases susceptibility to depression-like endophenotypes by chronic variable stress. Genes Brain Behav.

[CR77] Kirshenbaum GS, Burgess CR, Déry N, Fahnestock M, Peever JH, Roder JC (2014). Attenuation of mania-like behavior in Na^+^, ^K^+-ATPase a3 mutant mice by prospective therapies for bipolar disorder: Melatonin and exercise. Neuroscience.

[CR78] Komiyama Y, Nishimura N, Dong XH, Hirose S, Kosaka C, Masaki H, Masuda M, Takahashi H (2000). Liquid chromatography mass spectrometric analysis of ouabain like factor in biological fluid. Hypertens Res.

[CR79] Krenn L, Kopp B (1998). Bufadienolides from animal and plant sources. Phytochemistry.

[CR81] Li R, El-Mallakh RS (2004). Differential response of bipolar and normal control lymphoblastoid cell sodium pump to ethacrynic acid. J Affect Disord.

[CR82] Li R, El-Mallakh RS, Harrison L, Changaris DG, Levy RS (1997). Lithium prevents ouabain-induced behavioral changes: Toward an animal model for manic-depression. Mol Chem Neuropathol.

[CR83] Liang M, Cai T, Tian J, Qu W, Xie ZJ (2006). Functional characterization of Src-interacting Na/K-ATPase using RNA interference assay. J Biol Chem.

[CR84] Lichtstein D, Samuelov S, Bourrit A (1985). Characterization of the stimulation of neuronal Na^+^, K^+^-ATPase activity by low concentrations of ouabain. Neurochem Int.

[CR85] Lichtstein D, Gati I, Samuelov S, Berson D, Rozenman Y, Landau L, Deutsch J (1993). Identification of digitalis-like compounds in human cataractous lenses. Eur J Biochem.

[CR86] Lichtstein D, Steinitz M, Gati I, Samuelov S, Deutsch J, Orly J (1998). Biosynthesis of digitalis-like compounds in rat adrenal cells: hydroxycholesterol as possible precursor. Life Sci.

[CR87] Lichtstein D, Ilani A, Rosen H, Horesh N, Singh SV, Buzaglo N, Hodes A (2018). Na^+^, ^K^+-ATPase signaling and bipolar disorder. Int J Mol Sci.

[CR88] Looney SW, El-Mallakh RS (1997). Meta-analysis of erythrocyte Na, K-ATPase activity in bipolar illness. Depres Anxiety.

[CR89] Mack A, Gao Y, Kakar S, Ratajczak M, El-Mallakh RS (2019). Review of animal models of bipolar disorder that alter ion regulation. Neurosci Biobehav Rev.

[CR90] Madan N, Xu Y, Duan Q, Banerjee M, Larre I, Pierre SV, Xie Z (2017). Src-independent ERK signaling through the rat α3 isoform of Na/K-ATPase. Am J Physiol Cell Physiol.

[CR91] Manunta P, Hamilton J, Rogowski AC, Hamilton BP, Hamlyn JM (2000). Chronic hypertension induced by ouabain but not digoxin in the rat: antihypertensive effect of digoxin and digitoxin. Hypertens Res.

[CR92] Manunta P, Hamilton BP, Hamlyn JM (2006). Salt intake and depletion increase circulating levels of endogenous ouabain in normal men. Am J Physiol Regul Integr Comp Physiol.

[CR93] Moorhead TWJ, McKirdy J, Sussmann JED, Hall J, Lawrie SM, Johnstone EC, Mcintosh AM (2007). Progressive gray matter loss in patients with bipolar disorder. Biol Psychiatry.

[CR95] Mynett-Johnson L, Murphy V, McCormack J, Shields DC, Claffey E, Manley P, McKeon P (1998). Evidence for an allelic association between bipolar disorder and a Na^+^, K^+^ adenosine triphosphatase alpha subunit gene (*ATP1A3*). Biol Psychiatry.

[CR96] Nestler EJ, Peña CJ, Kundakovic M, Mitchell A, Akbarian S (2016). Epigenetic basis of mental illness. Neuroscientist.

[CR97] Noël F, Azalim P, do Monte FM, Quintas LEM, Katz A, Karlish SJD (2018). Revisiting the binding kinetics and inhibitory potency of cardiac glycosides on Na+, K+-ATPase(α1β1): Methodological considerations. J Pharmacol Toxicol Methods.

[CR98] Norn S, Kruse PR. Cardiac glycosides: From ancient history through Withering's foxglove to endogeneous cardiac glycosides [Article in Danish]. Dan Medicinhist Arbog 2004:119–132. 15685783

[CR99] Nurnberger J, Jimerson DC, Allen JR, Simmons S, Gershon E (1982). Red cell ouabain-sensitive Na+-K+-adenosine triphosphatase: a state marker in affective disorder inversely related to plasma cortisol. Biol Psychiatry.

[CR100] Overstreet DH (1993). The Flinders Sensitive Line Rats: A genetic animal model of depression. Neurosci Biobehav Rev.

[CR102] Philibert RA, Cheung D, Welsh N, Damschroder-Williams P, Thiel B, Ginns EI, Gershenfeld HK (2001). Absence of a significant linkage between Na^+^, ^K^+-ATPase subunit *(ATP1A*3 and* ATP1B*3) genotypes and bipolar affective disorder in the Old-Order Amish. Am J Med Genet.

[CR103] Pierre SV, Sottejeau Y, Gourbeau JM, Sánchez G, Shidyak A, Blanco G (2008). Isoform specificity of Na-K-ATPase-mediated ouabain signaling. Am J Physiol Renal Physiol.

[CR104] Rice D (1956). The use of lithium salts in the treatment of manic states. J Ment Sci.

[CR105] Roberts RJ, Repass R, El-Mallakh RS (2010). Effect of dopamine on intracellular sodium: a common pathway for pharmacologic mechanism of action in bipolar illness. World J Biol Psychiatry.

[CR106] Rose AM, Mellett BJ, Valdes R, Kleinman JE, Hermann MM, Li R, El-Mallakh RS (1998). Alpha2 isoform of the Na, K-ATPase is reduced in temporal cortex of bipolar individuals. Biol Psychiatry.

[CR107] Rosenblat JD, McIntyre RS (2016). Bipolar disorder and inflammation. Psychiatr Clin North Am.

[CR108] Saunders R, Scheiner-Bobis G (2004). Ouabain stimulates endothelin release and expression in human endothelial cells without inhibiting the sodium pump. Eur J Biochem.

[CR109] Schou M, Juel-Nielsen N, Stromgren E, Voldby H (1954). The treatment of manic psychoses by the administration of lithium salts. J Neurol Neurosurg Psychiatry.

[CR110] Schroeder E, Gao Y, Lei Z, Roisen F, El-Mallakh RS (2016). The gene *BRAF* is underexpressed in bipolar subject olfactory neuroepithelial progenitor cells undergoing apoptosis. Psychiatry Res.

[CR111] Shah A, Alshaher M, Dawn B, Siddiqui T, Longaker RA, Stoddard MF, El-Mallakh RS (2007). Exercise tolerance is reduced in bipolar illness. J Affect Disord.

[CR112] Shaw DM (1966). Mineral metabolism, mania, and melancholia. Br Med J.

[CR113] Skou JC (1957). The influence of some cations on an adenosine triphosphatase from peripheral nerves. Biochim Biophys Acta.

[CR115] Takaesu Y (2018). Circadian rhythm in bipolar disorder: A review of the literature. Psychiatry Clin Neurosci.

[CR116] Thomas SM, Brugge JS (1997). Cellular functions regulated by Src family kinases. Annu Rev Cell Dev Biol.

[CR117] Touza NA, Pôças ES, Quintas LE, Cunha-Filho G, Santos ML, Noël F (2011). Inhibitory effect of combinations of digoxin and endogenous cardiotonic steroids on Na^+^/K^+^-ATPase activity in human kidney membrane preparation. Life Sci.

[CR118] Valdes R, Hagberg JM, Vaughan TE, Lau BWC, Seals DR, Ehsani AA (1988). Endogenous digoxin-like immunoreactivity in blood is increased during prolonged strenuous exercise. Life Sci.

[CR119] Valvassori SS, Dal-Pont GC, Resende WR, Varela RB, Lopes-Borges J, Cararo JH, Quevedo J (2019). Validation of the animal model of bipolar disorder induced by Ouabain: face, construct and predictive perspectives. Transl Psychiatry.

[CR122] Yuan P, Zhou R, Wang Y, Li X, Li J, Chen G, Guitart X, Manji HK (2010). Altered levels of extracellular signal-regulated kinase signaling proteins in post- mortem frontal cortex of individuals with mood disorders and schizophrenia. J Affect Disord.

